# Evaluation of Different Risk Factors for Metastatic Sentinel Lymph Nodes in Endometrial Cancer

**DOI:** 10.3390/cancers16234035

**Published:** 2024-12-01

**Authors:** Michele Peiretti, Alfonso Altieri, Giorgio Candotti, Giuseppina Fais, Andrea Ungredda, Valerio Mais, Daniela Fanni, Stefano Angioni

**Affiliations:** 1 Department of Surgical Sciences, Division of Gynaecology and Obstetrics, University of Cagliari, 09042 Cagliari, Italy; mpeiretti@aoucagliari.it (M.P.); g.fais1@studenti.unica.it (G.F.); a.ungredda@studenti.unica.it (A.U.); valerio.mais@unica.it (V.M.); sangioni@unica.it (S.A.); 2 Department of Obstetrics and Gynecology, IRCCS Ospedale San Raffaele, 20132 Milan, Italy; candotti.giorgio@hsr.it; 3 Department of Medical Sciences, Pathology Unit, University of Cagliari, 09124 Cagliari, Italy; daniela.fanni@unica.it

**Keywords:** endometrial cancer, sentinel lymph node, macrometastasis, micrometastasis, isolated tumor cells

## Abstract

*Background/Objectives*: This study investigates which demographic, clinical and pathological factors of women with early-stage presurgical EC could be considered risk factors for the presence of different subtypes of metastases in sentinel lymph nodes (SLNs). *Methods*: This is a retrospective single-center study that collected data between December 2015 and April 2024. EC patients who underwent total hysterectomy with salpingo-oophorectomy and SLN mapping with indocyanine green (ICG) were recorded. *Results*: Data from 98 women with EC were analyzed. The endometrioid histotype was present in 85 (86%) women, and the non-endometrioid histotype was present in 13 (13%) women. High-grade EC (G3) was present in 21 (21.4%) patients, and low-grade EC (G1-G2) was present in 77 (78.6%) patients. The total number of women with SLN metastasis was 21/98 (21%). Of 21 women, 5 had MAC, 6 had MIC and 10 had ITCs. *Conclusions*: Preliminary analysis identified three risk factors for nodal involvement: age greater than 67 years, high-grade endometrial carcinomas and myometrial invasion greater than or equal to 50%. Lymphovascular space invasion, histotype 2 and p53 mutation showed a slight, but not statistically significant, tendency to be risk factors for SLN positivity. A deeper analysis with univariate uninominal logistic regression showed that high-grade EC is related to a greater probability of MACs, as shown in other studies, and that low-grade EC (grades 1 and 2) had a strong relationship with low-volume metastasis (LVM); further studies are needed to confirm these results.

## Simple Summary

Risk factors for nodal involvement, one of the most important prognostic factors in endometrial cancer (EC), have been widely studied, but to our knowledge, no studies have compared endometrial cancer risk factors among macrometastasis (MAC), micrometastasis (MIC) and isolated tumor cells (ITCs) in this selected lymphatic staging cohort. Our study evaluates clinical and pathological data of patients with presurgical early-stage endometrial carcinoma staged with SLN biopsy and their significance as risk factors for any subtype of nodal metastasis.

## 1. Introduction

Endometrial cancer has a global incidence of 8.7 per 100,000, and it is the seventeenth most common malignancy in the world and the fourth most common malignant neoplasm in the female population of the European Union. According to the “Annual Report to the Nation on the status of cancer: part I”, endometrial cancer was diagnosed in 417,367 women in 2020, worldwide. In 2021, the number of endometrial cancer-related deaths was 3100, and the most up-to-date estimated survival rate at 5 years after a diagnosis of endometrial cancer is 79% [1,2].

More than 90% of endometrial cancer cases are diagnosed in elderly individuals. The majority of endometrial cancer cases are sporadic, while approximately 5% of them are caused by genetic syndromes, such as Lynch syndrome or hereditary nonpolyposis colorectal cancer (HNPCC) syndrome.

Surgical staging of apparent early-stage endometrial cancer includes total hysterectomy with bilateral salpingo-oophorectomy and pelvic lymph node dissection (PLND) with or without para-aortic lymph node dissection [3].

Two important randomized trials demonstrated that systematic PLND had no impact on survival rates but seemed to maintain its role in defining patient prognosis [4,5]. Furthermore, systematic lymphadenectomy exposes patients to early or late complications, such as lower-extremity lymphedema [6]. A breakthrough in staging was achieved by the sentinel lymph node (SLN) technique, especially after the FIRES trial, which proved its comparability with systematic PLND for staging lymph node disease involvement, resulting in fewer complications [7]. Thus, SLN biopsy plays a key role in the staging of low- to intermediate-risk endometrial cancer, and in the last few years, it has been proven to be a valid alternative in the hands of experienced surgeons, even for high-to-intermediate-risk or high-risk disease [8]; however, some surgical techniques and the presence of enlarged nodes can affect the detection of SLNs [9].

Identified SLNs should be subjected to extensive pathological evaluation called “ultrastaging” [10], which seems to detect significantly more micrometastases (MICs, 0.2–2 mm) and isolated tumor cells (ITCs, ≤0.2 mm), defined as low-volume metastases (LVMs), than the classic analysis, which can instead detect macrometastases (MACs) well.

ITCs are often found incidentally during detailed examination and are generally considered less concerning than micrometastasis or macrometastasis. In endometrial cancer, ITCs may have a limited impact on staging and are sometimes regarded as having minimal clinical significance compared to larger metastatic deposits, though they still indicate that cancer cells have exited the primary site.

Although micrometastases are less extensive than macrometastases, their presence still indicates that cancer has spread beyond the primary tumor site. Macrometastases instead indicate more significant lymph node involvement, often suggesting a more advanced stage of disease and potentially indicating a need for more aggressive treatment approaches, such as systemic therapy.

Moreover, the “ultrastaging” and pathologic review of negative pelvic lymph nodes of patients with presumed isolated para-aortic metastasis from EC can identify occult pelvic dissemination and reduce the prevalence of true isolated para-aortic disease [11].

This retrospective study aimed to evaluate the clinical and pathological data of patients with presurgical early-stage endometrial carcinoma staged with SLN biopsy and their significance as risk factors for any subtype of nodal metastasis. Risk factors for nodal involvement, one of the most important prognostic factors in endometrial cancer, have been widely studied, but to our knowledge, no studies have compared endometrial cancer risk factors among ITCs, MICs and MACs in this selected lymphatic staging cohort.

## 2. Materials and Methods

This was an observational retrospective single-center study approved by the IRB (NP/2023/2538). We reviewed all patients with a diagnosis of endometrial cancer who underwent total hysterectomy with salpingo-oophorectomy and SLN mapping with indocyanine green (ICG) and had at least a successful unilateral SLN biopsy at our institution between December 2015 and April 2024. The surgical procedures were performed by two primary surgeons (M.P. and S.A.).

Different data were collected through our internal database. These data included: age and body mass index (BMI); tumor markers as well as Cancer Antigen 125 (CA125), Cancer Antigen 19-9 (CA 19-9) and Human Epididymis Protein 4 (HE-4), all sampled within 30 days prior to surgery; International Federation of Gynaecology and Obstetrics (FIGO) stage, tumor grade and histotype; pathological features like myometrial invasion (MI), lymphovascular space invasion (LVSI), tumor protein 53 (p53) and marker of proliferation Ki-67 (Ki-67); immunohistochemistry including L1 Cell Adhesion Molecule (L1CAM), estrogen receptor (ER), progesterone receptor (PR); and lymph nodal status with the type of lymph node metastasis (LNM), ITC, MIC or MAC.

### 2.1. Surgical Procedure

After a thorough preoperative evaluation, which included imaging studies with CT scans of the chest, abdomen and pelvis, as well as an endometrial biopsy, the surgical risk assessment was conducted.

Based on the preoperative risk factors, a laparoscopic or laparotomy approach was chosen in favor of the less risky route for each patient. The following steps were the same for both approaches. The ICG injection site was the cervix, 0.5 ccs submucosally, and 0.5 ccs deep 1 cm in the stroma at 3 and 9 o’clock with a total of 2 mL of ICG dye. The use of the near-infrared (NIR) mode allows the identification of pelvic SLNs that are excised. Then, a total hysterectomy was performed, and if SLNs were not identified and the clinical condition of the patient was adequate, an ipsilateral pelvic systematic lymphadenectomy was performed. All anatomical specimens were sent for final pathology. If the SLNs were confirmed to be lymph nodes with hematoxylin-and-eosin staining, they were submitted to an expert gynecologic pathologist (D.F.) for ultrastaging analysis.

### 2.2. Statistical Analysis

The data were organized in a Microsoft Excel [12] datasheet, and statistical analysis was performed by Wolfram’s Mathematica [13].

Clinical and pathological characteristics are summarized using basic descriptive statistics. Categorical and ordinal variables are described as absolute and relative frequencies. Continuous variables are expressed as the median (min–max).

We analyzed the correlation between different risk factors and metastatic SLNs with odds ratio calculations. For continuous variables without a pathological cutoff, such as age and BMI, we subdivided the patients into equally distributed groups by the median value. We also used an arbitrary cutoff for immunohistochemistry percentage (ER, PR and Ki-67). For tumor markers (CA125, CA 19-9 and HE4), we used our laboratory’s cutoffs that we recently aligned with other important Italian centers (especially HE4) [14].

Unifactorial and multifactorial risk factor analyses were performed on variables that were statistically significant or slightly correlated with SLN metastases in the odds ratio analysis. According to this test’s characteristics, variables with incomplete data were excluded. We used uninominal logistic regression to identify independent risk factors regardless of LNM size. Multinomial logistic regression, first subdivided into the MAC and LVM groups and then into the three types of metastases, was used to identify the impact of risk factors on the three subtypes of metastases. Additionally, multivariate analysis was performed.

## 3. Results

We selected 98 patients who underwent SLN mapping with ICG dye intracervical injection during laparoscopic (94%) or laparotomic (4%) hysterectomy for endometrial cancer. All clinical, surgical and pathological data are summarized in Table 1.

The median age at surgery was 67 years (32 to 88 years), and the median BMI was 28.1 kg/m^2^ (18.7 to 44 kg/m^2^). Regarding histotypes, 85 out of 98 patients (87%) had the endometrioid subtype, while 13 out of 98 (13%) presented with the non-endometrioid subtype (type 2). Final pathology results revealed that 77 out of 98 patients (77%) had low-grade endometrial carcinomas (G1–G2) whereas 21 out of 98 (21%) had high-grade endometrial carcinomas.

After pathological assessment, sentinel lymph nodes (SLNs) were negative in 77 out of 98 patients (79%). A total of 21 out of 98 patients (21%) exhibited SLN metastases. For patients who had more than one type of metastasis, we filtered by the worst subtype of LNM. Among these, 6 out of 21 patients (28%) had macrometastases (MACs), while 15 out of 21 (71%) had low-volume metastases. Of the latter, 5 out of 21 patients (24%) exhibited micrometastases (MICs), and 10 out of 21 (48%) had isolated tumor cells (ITCs). 

An odds ratio analysis, summarized in Table 2, identified age ≥67 years, high-grade endometrial carcinomas, and ≥50% myometrial invasion as significant risk factors for LNMs. Conversely, estrogen receptor expression >70% appeared to serve as a protective factor against nodal metastases. Additionally, mutated p53, lymphovascular space invasion (LVSI), and type 2 endometrial carcinoma histotypes demonstrated a slight but statistically non-significant correlation with LNMs. 

As shown in Table 3, simple logistic regression of the suggested risk factors revealed that myometrial invasion greater than or equal to 50% had a statistically significant relationship with LNMs, while age, grading and histotype had a weak linear relationship with nodal involvement.

At the same time, multivariate uninominal logistic regression analysis revealed that none of the variables considered were significant independent predictors of LNMs.

After univariate multinomial regression, we found that high-grade EC (grade 3) decreases the odds of LVMs compared with MACs in a statistically significant manner (*p* value = 0.047), as summarized in Table 4. At the same time, low-grade EC (grades 1 and 2) had a relationship with LVMs.

As shown in Table 5, simple logistic regression of the suggested risk factors for nodal involvement by nodal metastasis type revealed that age and high-grade carcinomas had a slight, but not significant, correlation with MACs. Moreover, the presence of ITCs in SLNs is more frequent in type 1, 10/21 (47.6%), and in G1–2, 8/21 (38%).

## 4. Discussion

In this retrospective study, we evaluated SLN detection rates and the possible risk factors for LNM in univariate and multivariate analyses.

The use of SLN biopsy in apparent early-stage endometrial cancer is based on strong scientific data. Its use in endometrial cancer was proposed by Burke et al. in 1996 [15], and since then it has been of increasing importance [8]. Following this trend, in 2015, we started to perform SLN biopsy in addition to hysterectomy and salpingo-oophorectomy because there was strong evidence that lymphadenectomy in early-stage endometrial cancer is not related to a better overall survival rate but remains an important staging tool [4,5].

In the last decade, the switch from demolitive to targeted lymphatic surgical staging has been prominent. The 2021 ESGO/ESTRO/ESP guidelines for the management of endometrial cancer indicate the SLN technique as the minimally invasive treatment of choice for lymph node staging [8]. In particular, the 2019 SHREC trial by J. Persson et al. demonstrated the diagnostic accuracy of the SLN technique in high-risk endometrial cancer [16].

It has been demonstrated that SLN mapping evaluation unveils a relevant proportion of metastases that traditional methods do not detect, with important implications for staging, tumor risk assessment and the necessity of adjuvant treatment [17]. Although the prognostic significance of ITCs is not yet clear, it has been shown that the presence of MICs is related to a slightly worse prognosis and that patients with MICs subjected to adjuvant treatment (Adx) have a better prognosis than patients treated only with surgery [18]. Moreover, not all patients with LVMs have the same prognosis, as shown by Ghoniem K., who found that LVSI, non-endometrioid histology and uterine serosae invasion were independent risk factors for recurrence in patients with these metastases [19].

In 2014, a retrospective study based on the US National Cancer Institute’s Surveillance, Epidemiology, and End Results (SEER) registry that considered women with endometrial cancer limited to the corpus uteri or lymph node identified correlations between tumor size, depth of invasion, histologic grade and lymph node involvement [20]. In a retrospective study of risk factors for pelvic LNM in EC after hysterectomy conducted by Li Y. et al. in 2019, BSO, pelvic lymphadenectomy, elevation of preoperative CA125, HE4, non-endometrioid histology, MI > or = to 50%, positive peritoneal cytology, LVSI, tumor size, depth of invasion and histologic grade were independent risk factors for overall pelvic LNMs [21]. Recently, endometrial cancer molecular subtypes, described in molecular classification [22,23], and LNMs showed a statistically significant association, revealing possible implications for lymph node staging planning [24].

In our study, preliminary analysis identified three risk factors for nodal involvement: age greater than 67 years, high-grade endometrial carcinomas and myometrial invasion greater than or equal to 50%. LVSI, histotype 2 and p53 mutation showed a slight, but not statistically significant, tendency to be risk factors for SLN positivity. Meanwhile, a higher percentage of ER was a protective factor for LNMs.

Univariate multinomial logistic regression showed that grade 3 endometrial cancer compared with grade 1 and grade 2 decreases the probability of LVMs related to MACs, and grade 3 endometrial cancer is significantly correlated with a lower probability of ITCs than MACs. In other words, if there is a metastasis in grade 3 endometrial cancer, it is more likely to be an MAC than an ITC. Supporting our results, in other studies, despite the analysis of slightly different populations, LVMs were found more frequently in low-grade and type 1 endometrial cancers [25,26].

According to a recent study published by Cucinella G et al., patients with SLN-isolated tumor cells and a low risk profile who did not undergo adjuvant therapy had significantly worse recurrence-free survival compared with node-negative patients with similar risk factors, after adjusting for grade and excluding patients with lymphovascular space invasion [27].

In our series, multivariate multinomial logistic regression did not show any difference between risk factors for MACs, MICs and ITCs. This could mean that the analyzed factors contribute in similar ways to the presence of MACs and LVMs. Another possibility is that our cohort was too small to perform an effective multifactorial multinomial analysis, as suggested by a recent study that analyzed the risk factors for LVMs in all risk classes of endometrial cancer treated with SLN mapping and found that MI greater than 50% was the only independent risk factor [26].

Our results also showed that estrogen receptor levels greater than 70% appeared to be a protective factor for nodal metastases, as already demonstrated by Ballester M. in 2013 [28]. The limitations of our study are the retrospective single-center design and the small sample size.

## 5. Conclusions

Our analysis showed that some variables could be considered risk factors for LNMs. Risk factors seem to be different for different subtypes of lymph nodal involvement. In our series, if a low-grade tumor has LNMs, they are more often LVMs. These data could be considered for more accurate communication with patients and to define the necessity for Adx therapy. LVMs’ role in recurrence risk has yet to be determined, as stated in a recent work by Fumagalli D. et al. [29]. Nevertheless, prospective and multicentric studies that employ the new molecular classification to better assess risk factors for the different subtypes of metastases should be performed.

## Figures and Tables

**Table 1:** Demographic, clinical and pathological characteristics of patients with endometrial cancer at permanent pathology included in the retrospective analysis.

Variables	Median (Range) or N (%)
Age (years)	67 (32–88)
BMI (kg/m^2^)	28.1 (18.7–44)
CA125 (U/L)	14 (5–169)
CA 19-9 (U/L)	16 (1–708)
HE4 (pmol/L)	103 (32–859)
Laparotomy	4 (4.1%)
Laparoscopy	94 (95.9%)
Histotype	
Type 1	85 (86.7%)
Type 2	13 (13.3%)
Grade	
1	50 (51%)
2	27 (27.6%)
3	21 (21.4%)
Myometrial invasion	
> or = 50%	38 (38.8%)
<50%	60 (61.2%)
LVSI	
Positive	13 (15,5%)
Negative	71 (84.5%)
p53 mutation	
NA	21 (21.4%)
Not valuable	23 (23.5%)
Aberrant	13 (13.3%)
Wild type	41 (41.8%)
L1CAM	
NA	33 (33.7%)
Positive	24 (24.5%)
Negative	41 (41.8%)
Receptors	
ER (%) median (range)	65 (0–95)
PR (%) median (range)	40 (0–90)
Ki-67 (%) median (range)	30 (0–70)
FIGO stage	
IA	54 (55.1%)
IB	28 (28.6%)
II	3 (3%)
IIIA	2 (2%)
IIIC	11 (11.2%)
Lymph node status	
Negative	77 (78.6%)
Empty nodes	0 (%)
ITCs	10 (10.2%)
MICs	5 (5.1%)
MACs	6 (6.1%)

BMI, body mass index; CA125, cancer antigen 125; CA 19-9, cancer antigen 19-9; HE4, Human Epididymis Protein 4; LVSI, lymphovascular space invasion; p53, tumor protein p53; NA, not available; L1CAM, L1 Cell Adhesion Molecule; ER, estrogen receptor; PR, progesterone receptor; Ki-67, marker of proliferation Ki-67; FIGO, International Federation of Gynaecology and Obstetrics; ITCs, isolated tumor cells; MICs, micrometastases; MACs, macrometastases.

**Table 2:** Impact of risk factors on nodal involvement—odds ratio assessment.

Factor	Positive Lymph Nodes	Odds Ratio			
	N (21)	CI− (95%)	OR	CI+ (95%)	*p* Value
Age ≥ 67 years	15	1.052	3	8.553	**0.030**
Age < 67 years	6				
0.BMI ≥ 27.8 kg/m^2^	10				0.464
BMI < 27.8 kg/m^2^	11				
CA125 ≥ 35 UI/mL	2				0.631
CA125 < 35 UI/mL	11				
CA 19-9 ≥ 39 UI/mL	2				0.382
CA 19-9 < 39 UI/mL	12				
HE4 ≥ 135 ng/mL	6				0.352
HE4 < 135 ng/mL	7				
Type 2 EC	5				0.110
Type 1 EC	16				
Grade = 3	8	1.046	3	8.776	**0.004**
Grade < 3	13				
Myometrial invasion +	12	1.012	2.6	7.003	**0.046**
Myometrial invasion −	9				
LVSI +	4				0.281
LVSI −	17				
p53 aberrant	5				0.179
p53 not valuable or wild type	14				
L1CAM +	6				0.237
L1CAM −	6				
ER ≥ 70%	7				0.08
ER < 70%	13				
PR ≥ 40%	9				0.294
PR < 40%	10				
Ki-67 ≥ 30%	11				0.575
Ki-67 < 30%	6				

N, number of cases; CI− or +, confidence interval negative or positive; OR, odds ratio; BMI, body mass index; CA125, cancer antigen 125; CA 19-9, cancer antigen 19-9; HE4, Human Epididymis Protein 4; LVSI, lymphovascular space invasion; p53, tumor protein p53; L1CAM, L1 Cell Adhesion Molecule; ER, estrogen receptor; PR, progesterone receptor; Ki-67, marker of proliferation Ki-67.

**Table 3:** Comparison of risk factors for nodal involvement—univariate and multivariate uninominal logistic regression analyses.

	LN+	Univariate Uninominal Log Regression	Multivariate Uninominal Log Regression
Factor	N (21)	*p* Value	*p* Value
Type 2	5	0.110	0.838
Type 1	16		
Age ≥ 67 y	15	0.035	0.012
Age < 67 y	6		
G 3 (14)	8	0.036	0.031
G 1–2 (44)	13		
MI > 50%	12	0.052	0.645
MI < 50%	9		

LN+, positive lymph node; Log, logistic; N, number of cases; G, grade; MI, myometrial invasion.

**Table 4:** Comparison of risk factors for nodal involvement by nodal macrometastases and low-volume metastases—univariate multinomial logistic regression analyses.

	MACs	LVMs	Univariate Multinomial Log Regression	Multivariate Multinomial Log Regression
Factor	N (5)	N (16)	*p* Value	*p* Value
Type 2	3	2	0.063	0.030
Type 1	2	14		
Age ≥ 67 y	5	10	0.148	0.116
Age < 67 y	0	6		
G 3	4	4	0.047	0.027
G 1–2	1	12		
MI ≥ 50%	3	9	0.647	0.890
MI < 50%	2	7		

MACs, macrometastases; LVMs, low-volume metastases; Log, logistic; N, number of cases; G, grade; MI, myometrial invasion.

**Table 5:** Comparison of risk factors for nodal involvement by nodal metastasis type (MACs, MICs and ITCs)—univariate multinomial logistic regression analyses.

	MACs	MICs	ITCs	Univariate Multinomial Log Regression	Multivariate Multinomial Log Regression
Factor	N (5)	N (6)	N (10)	*p* Value ^A^	*p* Value ^A^
Type 2	3	2	0	0.010	0.025
Type 1	2	4	10		
Age ≥ 67 y	5	5	5	0.038	0.104
Age < 67 y	0	1	5		
G 3	4	2	2	0.035	0.079
G 1–2	1	4	8		
MI ≥ 50%	3	5	4	0.324	0.263
MI < 50%	2	1	6		

MACs, macrometastases; MICs, micrometastases; ITCs, isolated tumor cells; Log, logistic; N, number of cases; G, grade; MI, myometrial invasion. ^A^: The value on the left refers to MICs compared to MACs and that on the right refers to ITCs compared to MACs.

## Data Availability

Data are contained within the article.
